# Observable impairments predict mortality of captured and released sockeye salmon at various temperatures

**DOI:** 10.1093/conphys/cou029

**Published:** 2014-08-14

**Authors:** Marika Kirstin Gale, Scott G. Hinch, Steven J. Cooke, Michael R. Donaldson, Erika J. Eliason, Ken M. Jeffries, Eduardo G. Martins, David A. Patterson

**Affiliations:** 1 Pacific Salmon Ecology and Conservation Laboratory, Department of Forest and Conservation Sciences, University of British Columbia, 2424 Main Mall, Vancouver, British Columbia, Canada V6T 1Z4; 2 Fish Ecology and Conservation Physiology Laboratory, Ottawa-Carleton Institute of Biology and Institute of Environmental Sciences, Carleton University, Ottawa, Ontario, Canada K1S 5B6; 3 Department of Natural Resources and Environmental Sciences, University of Illinois at Urbana-Champaign, 1102 S. Goodwin Ave., Urbana, IL 61801, USA; 4 Anatomy, Physiology & Cell Biology, School of Veterinary Medicine, University of California, Davis, One Shields Avenue, Davis, CA 95616, USA; 5 Cooperative Resource Management Institute, Fisheries and Oceans Canada, School of Resources and Environmental Management, Simon Fraser University, TASC 1 - Room #8405, 8888 University Drive, Burnaby, British Columbia, Canada V5A 1S6

**Keywords:** Fisheries, mortality, stress, temperature

## Abstract

Migrating adult sockeye salmon frequently encounter commercial and recreational fishing gear, from which they may be landed, escape or be intentionally released. In this experiment, migratory adult sockeye salmon were exposed to simulated capture–release in fresh water, including 3 min of exhaustive exercise and 60 s of air exposure at three ecologically relevant water temperatures (13, 16 and 19°C) to understand how thermal and capture–release stressors may interact to increase mortality risk. Water temperature and sex were the factors that best predicted 24 and 48 h survival, with females in the warmest temperature group experiencing the greatest mortality. Capture–release treatment including air exposure was associated with equilibrium loss and depressed ventilation rates at release; the probability of fish surviving for 24 h after simulated capture–release was >50% if the duration of equilibrium loss was <2 min or ventilation frequency was >1 breath s^−1^. Higher haematocrit and plasma lactate as well as lower mean cell haemoglobin concentration and plasma sodium and chloride 30 min after simulated capture–release were also significant predictors of 24 h survival. Together, the results demonstrate that simple observations that are consistent with physiological disturbance can be used as predictors for post-release short-term survival for sockeye salmon. The markedly higher post-stressor mortality observed in females demonstrates that managers should consider sex-specific variation in response to different fisheries interactions, particularly in the face of climate change.

## Abstract (precis)

We investigated how water temperature and the stressors imposed by encounters with fishing gear (e.g. catch-and-release angling) interact to affect the physiology and survival of sockeye salmon during their upstream reproductive migration. We found that we could use simple observations such as breathing rate and righting response to predict whether individuals would survive after simulated catch-and-release.

## Introduction

In most fisheries, some fish escape or are released from gears, but the survival of these non-retained fish is highly uncertain. Volitional releases are typically based on the premise that fish are returned to the water in a manner such that they survive and reproduce (Wydoski *et al.*, 1976). However, mortality does occur, with estimates ranging from zero to almost 100% across different fisheries (Muoneke and Childress, 1994; Chopin and Arimoto, 1995; Davis, 2002; Bartholomew and Bohnsack, 2005; Arlinghaus *et al.*, 2007). Mortality is highly context dependent (Cooke and Suski, 2005) and varies by species, anatomical hooking location, capture depth, hook, bait or gear types, air exposure, life-history stage and/or size, handling and water temperature, with water temperature being a common feature in most studies (reviewed by Muoneke and Childress, 1994; Davis, 2002; Arlinghaus *et al.*, 2007). A recent review of the role of water temperature in capture–release fisheries revealed a paucity of knowledge about the effects of ecologically relevant high temperatures on the mortality and impairment of released fish (Gale *et al.*, 2013).

In British Columbia's Fraser River, sockeye salmon (*Oncorhynchus nerka*) are of particular interest in terms of capture–release at high temperatures. Fraser River summer water temperatures have increased by ∼2°C in the past 60 years, and in the last decade sockeye salmon have encountered the highest Fraser River water temperatures ever recorded (Patterson *et al.*, 2007). This is significant because Fraser River sockeye salmon stocks have adapted their aerobic capacity to be optimized at thermal environments historically experienced (Farrell *et al.*, 2008; Eliason *et al.*, 2011), and their physiological functioning becomes impaired at water temperatures beyond their optimum (Farrell, 2009). In years of high river temperatures, some late-run stocks have experienced en route migration mortalities of up to 90% (Cooke *et al.*, 2004, Hinch *et al.*, 2012).

Fraser River sockeye salmon are a highly valued resource. For example, out of the estimated total run size of 30 million in 2010 (DFO, 2011b), 10 million were caught by commercial fishers (DFO, 2011a), 1.2 million by aboriginal groups (DFO, 2010a) and 300 000 by recreational anglers (DFO, 2010b). For these fishing sectors, there are few estimates of how many fish are caught but escape gear prior to landing; however, large proportions of fish are seen on spawning grounds bearing the characteristic scars from their encounters with fishing gears (e.g. gill-net wounds and hook wounds; Clarke *et al.*, 1994). A similar phenomenon has been observed for Alaskan sockeye salmon (Baker and Schindler, 2009). Almost 33% of Fraser River sockeye caught by recreational anglers in 2010 were reported to be released (DFO, 2010b). Many of these fishery encounters occur during a particularly difficult portion of the adult life history, because fish have just transitioned from cool salt water to warm fresh water, ceased feeding, and must achieve remarkable feats of metabolic performance to ascend the river. Presently, we know little about the consequences of capture encounters on released or escaped sockeye salmon, particularly when they are experiencing thermal stress. The exceptions are a similar experiment to the present study, where capture stress caused summer-run sockeye salmon to exhibit greater physiological impairment in warmer water temperatures than in cooler ones (Gale *et al.* 2011), and a recent study (Robinson *et al.*, 2013) in which Fraser River sockeye that were exhausted following fishing simulations were exposed to facilitated recovery techniques. These authors conducted their work at two temperatures and revealed that facilitated recovery failed to reduce mortality.

Currently, in-season harvest adjustments are made by managers using highly uncertain and complex statistical models that predict the proportion of each run timing group that will be likely to perish en route as a result of forecasted water temperatures (e.g. Macdonald *et al.*, 2010; Cummings *et al.*, 2011). These models, designed to increase the probability of achieving spawning escapement goals, do not explicitly account for release mortality and/or the interaction of fish and fishing gear at warmer temperatures. Therefore, reducing the uncertainty in this management system by being able more accurately to predict and account for release and escape mortality at different temperatures can assist in the management of this important economic, cultural and ecological resource.

We simulated capture and release of adult sockeye salmon in a laboratory, which involved exhaustive exercise and air exposure at three different temperatures relevant to migrating Fraser River fish. These temperatures included a cool historic average (13°C), a moderate current average near the optimum for aerobic scope (16°C) and a current high near the critical thermal maximum for aerobic scope (19°C; Farrell *et al.*, 2008). We investigated the following hypotheses: (i) equilibrium and ventilation would be impaired following capture–release as stressors were incrementally added (i.e. exhaustive exercise, air exposure and higher water temperatures); (ii) physiological disturbances (i.e. changes in blood plasma ions and metabolites) would increase following capture–release as those stressors were incrementally added; and (iii) mortality would be highest in groups with the greatest cumulative stressors (i.e. fish exhaustively exercised and air exposed at the warmest temperature). We used our results to predict the probability of individual survival following capture–release using physiological metrics.

## Materials and methods

### Study animals and facility

**Figure 1:**
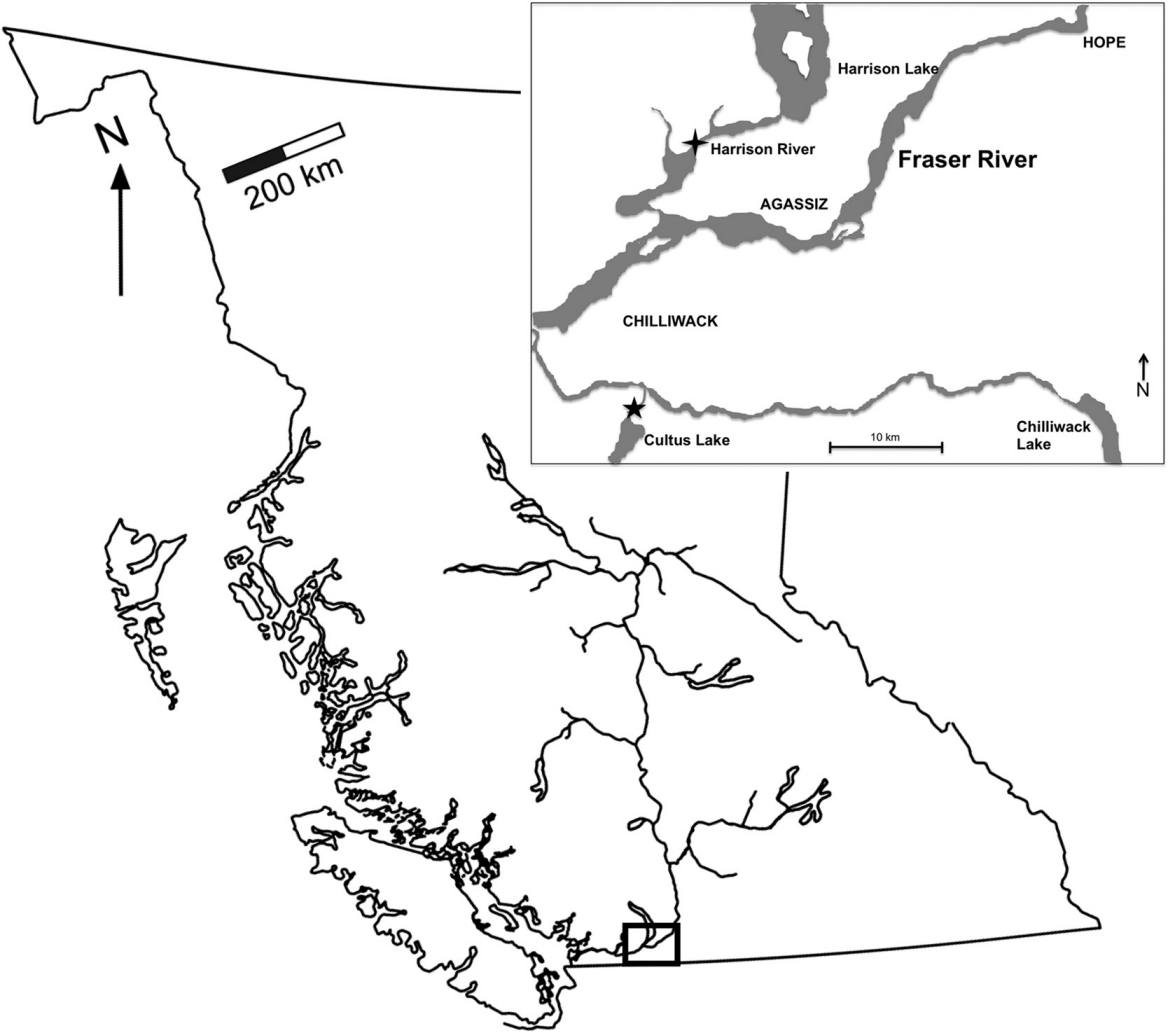
British Columbia and the Fraser River, which drains almost one-third of the province. Inset is the study area, with the cross marking the fish capture site on the Harrison River, and the star marking the Cultus Lake Salmon Research Laboratory, where experiments took place.

We intercepted adult sockeye salmon in British Columbia's Harrison River, a tributary of the Fraser River, located ∼125 km east of Vancouver (Fig. 1). These sockeye salmon were captured by beach seine, 15–18 September 2008, while completing their spawning migration, and belonged to the Weaver Creek population. Fish were individually dip-netted from the seine net, transferred to tanks (1000 l) mounted on trucks and then transported (∼45 min) to the Cultus Lake Salmon Research Laboratory (Cultus Lake, BC, Canada). Upon arrival, each sockeye salmon was sampled for adipose fin tissue in order to determine population identification. At this time, PIT tags (full-duplex Passive Integrated Transponder tags, ∼8.5 mm × 2 mm size, 134.2 kHz; Biomark Inc., Boise, ID, USA) were injected by 12 gauge needle into the coelomic cavity for individual identification. No anaesthesia or wound closure was necessary for this procedure. Fish were then introduced to a large (20 000 l, 6 m diameter) artificial holding pond fed continuously with ultraviolet-sterilized (∼40 l min^−1^; LS-Permabead Filtration System, Integrated Aqua Systems Inc., Escondido, CA, USA) 9°C water drawn from nearby Cultus Lake. On 22 September 2008, after receiving DNA results indicating the stock identity of each fish, 54 of 106 Weaver Creek sockeye salmon (a late-run stock) were moved into nine smaller (1400 l) aquaria (six or seven fish per tank) maintained at 11°C for 24 h. After this period of recovery from transport stress (based on previous experience with this species), we began increasing the water temperature (maximum rate, 0.2°C h^−1^) until experimental temperatures were achieved (three tanks each at 13, 16 and 19°C). A minimum of 12 h elapsed prior to initiating experimental fishing simulations. Although that time period may appear short, previous field work on sockeye salmon has revealed that they experience massive thermal variation over short temporal scales during homeward migration (e.g. >10°C over 24 h; Drenner *et al.*, 2014). Due to logistical constraints, the experimental treatments were applied in two consecutive rounds, with 54 fish in the first round of the experiment, after which they were removed from the treatment tanks and placed back in the large holding ponds to allow for the experiment to be run on another 52 fish. The two rounds were pooled for analyses.

### Experimental protocol

After all fish had been maintained at their treatment temperatures for a minimum of 12 h, they were randomly assigned to one of three simulated capture–release treatments, resulting in a full-factorial design using three levels of temperature and three levels of capture–release stress. The lowest magnitude stressor involved handling only, in which individuals were netted from their holding tank, identified using a hand-held PIT reader (Biomark Inc.) and transferred to one of the treatment tanks (at the experimental temperature for each fish) and monitored for 30 min. This treatment was designed as a non-capture control for handling effects other than those employed in the two simulated capture–release treatments. The capture–release stressor included stimulating fish to burst swim for 3 min by manual chasing. This technique involved three experimenters standing around the perimeter of the treatment tank, leaning over and splashing vigorously behind the fish or gently touching its tail, and has been used extensively in angling simulation and exercise experiments (Milligan, 1996; Kieffer, 2000). The third treatment, capture plus air exposure, involved the capture–release stressor described above followed by gently lifting fish out of the water using a soft mesh dip-net to air expose them for 60 s.

Both groups of capture-stressed fish were blood sampled 30 min after the instigation of chasing. Blood was collected via caudal venipuncture while holding fish supine in a foam-lined trough supplied with flowing water. Transfer to the trough and blood sampling occurred quickly (<1 min) in order to reduce the influence of sampling stress on physiological test results (Cooke *et al.*, 2005, 2013). Fish were also marked by anchor tag and sampled at this time for a small gill sample (3 mm from the distal end of five to 10 filaments on the first gill arch on the left side of the fish) and 3 mm muscle punch for gene expression analysis in a separate experiment (Donaldson, 2012). These biopsy procedures have been previously shown to cause no alteration of behaviour or survival in adult sockeye salmon (e.g. Cooke *et al.*, 2005). No anaesthesia was used (as per Cooke *et al.*, 2005) given that fish were easy to handle when in a supine position with constant flow of aerated water. Moreover, anaesthesia would have introduced yet another potential physiological challenge from which fish would have to recover, which could confound experiments. Ventilation rates for all fish were counted immediately after introducing fish to the treatment tank, immediately after capture–release treatment with/without air exposure, and again before blood and tissue sampling (30 min later). Fish were also monitored for the presence or absence of the ability to maintain dorsoventral equilibrium post-capture–release, as well as the duration of this impairment. After sampling, fish were returned to their holding tanks. Overall, 47 female and 50 male sockeye salmon completed the experiment. Sockeye salmon that died prior to the simulated capture–release treatment were excluded from analyses.

Fish were monitored hourly for the duration of the experiment. Dead fish were removed from the tanks, and moribund fish (i.e. those that had lost equilibrium and demonstrated erratic or absent swimming behaviour) were removed and euthanized by cerebral concussion to comply with animal care protocols. Moribund fish were considered as ‘mortalities’ for analyses. All surviving fish were sampled 48 h after capture–release treatments. All experimental procedures were conducted with approval from the University of British Columbia Animal Care Committee (#A08-0388) and in accordance with guidelines set forth by the Canadian Council on Animal Care.

### Laboratory protocols and assays

Stock identification was determined for all individual fish using DNA analyses (Beacham *et al.*, 2004) and confirmed by analyses of scales. All 97 sockeye salmon used in the analysis were from the late-run Weaver Creek stock. Haematocrit was quantified on whole blood using microcapillary tubes centrifuged at 10 000*g* for 2 min. The remaining blood was centrifuged at 7000*g* for 6 min, and plasma was stored at −80°C until further analysis. Plasma was subsequently analysed for cortisol, lactate, glucose, osmolality, chloride, sodium and potassium, as described by Farrell *et al.* (2001). Briefly, plasma analysis was conducted using the following instruments: cortisol, Neogen ELISA with Molecular Devices Spectramax 240pc plate reader; lactate and glucose, YSI 2300 Stat Plus analyzer; osmolality, Advanced Instruments 3320 freezing-point osmometer; chloride, Haake Buchler digital chloridometer; and sodium and potassium, Cole-Parmer, model 410 single-channel flame photometer. Haemoglobin was measured using a hand-held haemoglobin meter (HemoCue 201 + , Angelholm, Sweden) following the protocol and calibration procedure described by Clark *et al.* (2008).

### Statistical analysis

All statistical analyses were performed using the R Statistical Package (R Development Core Team, 2008). Three-way ANOVA was used to detect differences in the responses of males and females to temperature and capture–release treatment. In the absence of a sex effect, two-way ANOVA was used to compare the duration of equilibrium loss, ventilation rates and individual plasma indices between capture–release treatment and temperature groups (including a temperature × capture–release treatment interaction), using log-transformations to reduce heteroscedasticity when necessary. Welch's *t*-tests were performed *post hoc* to determine where significant differences occurred. Pearson's χ^2^ tests were used to evaluate differences in the frequency of equilibrium loss between treatment groups. Significance for all analyses was evaluated at the level of α = 0.05, and multiple comparisons were corrected for using the false-discovery rate method, where each comparison is evaluated against the critical significance level, equal to the false-discovery rate of 0.05 multiplied by the number of the comparison divided by the total number of comparisons (Curran-Everett, 2000).

Survival analysis was performed using the ‘survival’ library (Therneau 2012) in R. Time to death (in hours over 48 h post-treatment) was analysed as a function of temperature, simulated capture–release treatment, sex and combinations of these (including two-way interactions). A model with no effects (i.e. intercept only) was also fitted to the time-to-death data. The 48 h time period was chosen for examination based on visualization and preliminary analyses of the survival results; mortality occurring after this period of time was considered to be unrelated to experimental treatments, instead occurring presumably due to the stressors of captivity and natural senescence. Model selection was carried out using the bias-corrected Akaike information criterion (AICc). According to this criterion, the model with the lowest AICc value is the most parsimonious one describing the data, and other models differing from this one in <2, 4–7 and >10 units (delta.AICc) are regarded as having substantial, considerably less and essentially no support from the data, respectively (Burnham and Anderson, 2002). The AICc weight of the models was also computed, and can be interpreted as the probability of a given model in the set being the most parsimonious one to describe the data (Burnham and Anderson, 2002). To account for model selection uncertainty, model-averaged mortality over time was computed using the AIC weight of the models included in a 95% confidence set for the best model (Burnham and Anderson, 2002). This resulted in a weighted combination of eight models being used to calculate model-averaged mortality.

Total osmolality values for four fish were unable to be determined by assay; therefore, the values were estimated using a linear regression that included the concentration of the major osmolites that contribute to total osmolality, resulting in the following equation:

$$\begin{array}{rr} \text{osmolality} & =(0.8)\times ([\text{lactate}]+[\text{glucose}]+[\text{sodium}]\\ & \;+[\text{chloride}]+[\text{potassium}])+73.1 \end{array}$$

where *n* = 92, *r*^2^ = 0.70 and *P* < 0.0001.

Logistic regression was used to test whether 24 h mortality (binary response) could be predicted by the fish's physiology (haematocrit, haemoglobin, plasma lactate, glucose, chloride, sodium and potassium concentration 30 min post-treatment), ventilation rate (opercular beats per minute) and duration of equilibrium loss (in seconds) immediately after release. These regressions were conducted separately for each of the above-mentioned variables and did not take into account the capture–release treatments (i.e. simply evaluated whether variability in physiological parameters, ventilation rate and equilibrium were associated with mortality regardless of the treatment). The fit of each model to the mortality data was assessed using the le Cessie–van Houwelingen–Copas goodness-of-fit tests implemented in the R library ‘rms’ (Harrell, 2013). The test computes a global goodness-of-fit test statistic based on the unweighted sum of squares of residuals (Harrell, 2001). The test has the advantage of being more powerful and not dependent on the choice of cut-off points for groups of predictions that is needed for the more commonly used Hosmer–Lemeshow test (Hosmer *et al.*, 1997). Given that logistic regression models were fitted separately to a number of predictor variables, multiple testing adjustments to critical values were done using the false-discovery rate method described previously (Curran-Everett, 2000).

## Results

### Observed impairments

Observed impairment was related to the combination of treatments applied. Air-exposed fish were more likely to lose equilibrium than non-air-exposed fish (Pearson χ^2^ = 74.41, *P* < 0.00001). For those fish that lost equilibrium after capture–release (31 of 32 air-exposed fish, three of 33 capture–release-only fish and none of 33 non-capture-treated fish), there was a significant effect of the temperature × treatment interaction on the duration of equilibrium loss (ANOVA: *F*_2,31_ = 11.15, *P* = 0.002). The longest equilibrium loss was observed in air-exposed fish at the warmest temperature. Ventilation rates measured after fish were transferred to the treatment tank were higher for 16 and 19°C fish than for 13°C fish (ANOVA: *F*_2,86_ = 4.53, *P* = 0.014). Immediately after simulated capture–release, temperature effects were no longer evident, but air-exposed fish were ventilating significantly more slowly than non-air-exposed fish (ANOVA: *F*_2,86_ = 4.67, *P* = 0.012).

### Blood chemistry

Blood chemistry disturbances 30 min post-capture–release were more strongly associated with simulated capture–release (i.e. exhaustive exercise) and air exposure than water temperature. Simulated capture–release significantly elevated plasma lactate concentration (Table 1; Welch's *t* = 5.62, *P* < 0.0001), with air exposure exacerbating this elevation (Table 1; Welch's *t* = 2.89, *P* = 0.005). Total osmolality was likewise increased by capture–release (Table 1; Welch's *t* = 2.97, *P* = 0.004) and air exposure (Table 1; Welch's *t* = 2.5, *P* = 0.015). Mean cell haemoglobin concentration (MCHC) was lowered by capture–release treatment (Table 1; Welch's *t* = 2.7, *P* = 0.009), but air exposure did not further depress MCHC (Table 1). Haematocrit tended to be higher in capture–release-treated individuals than in control fish, while haemoglobin concentration was similar among groups (Table 1). Water temperature had no effect on any plasma or blood variable we measured at 30 min (ANOVA: *P* > 0.1). Plasma glucose was not affected by capture–release treatment (Table 1). Plasma cortisol was higher for females than males, but capture–release treatment had no effect on cortisol levels for either sex (Table 1). Plasma chloride, sodium and potassium were also not different among capture–release treatment groups (Table 1). Blood chemistry tests were repeated on surviving fish at 48 h post-capture–release, at which time no effect of capture–release or temperature was detectable after correcting for multiple comparisons (data not shown).

**Table 1:** Mean (±SEM) plasma constituent concentrations for each capture-stressor group and temperature treatment group, measured from sockeye salmon 30 min after the application of a simulated capture–release stressor at 13, 16 or 19°C

Capture–release treatment	Mean ± SEM	Capture treatment		Temperature group (°C)	Mean ± SEM	Temperature	
		*F*	*P* value			*F*	*P* value
Chloride (mmol l^−1^)							
No exercise	130.00 ± 1.00	2.5	0.084	13	131.52 ± 1.34	1.2	0.86
Exercise	131.84 ± 1.48			16	131.61 ± 1.33		
Exercise + air	133.77 ± 1.19			19	132.40 ± 1.03		
Cortisol – females (ng ml^−1^)							
No exercise	291.58 ± 23.57	0.0068	0.99	13	271.32 ± 18.35	0.29	0.75
Exercise	313.87 ± 25.28			16	291.69 ± 28.65		
Exercise + air	271.40 ± 20.27			19	333.40 ± 21.47		
Cortisol – males (ng ml^−1^)							
No exercise	162.83 ± 12.68	0.49	0.61	13	143.16 ± 12.60	0.47	0.63
Exercise	165.59 ± 15.15			16	154.12 ± 15.12		
Exercise + air	156.62 ± 12.82			19	188.83 ± 9.33		
Glucose (mmol l^−1^)							
No exercise	6.22 ± 0.24	2.6	0.084	13	6.62 ± 0.29	0.38	0.69
Exercise	7.17 ± 0.29			16	6.93 ± 0.27		
Exercise + air	7.28 ± 0.30			19	7.15 ± 0.31		
Lactate (mmol l^−1^)							
No exercise	5.60 ± 0.49^a^	10.8	** < 0.00001**	13	8.49 ± 0.69	0.82	0.44
Exercise	9.75 ± 0.55^b^			16	9.22 ± 0.71		
Exercise + air	12.3 ± 0.69^c^			19	9.82 ± 0.87		
Osmolality (mosmol kg^−1^)							
No exercise	311.12 ± 2.21^a^	6.8	**0.0019**	13	320.79 ± 2.86	0.15	0.86
Exercise	321.73 ± 2.80^b^			16	319.52 ± 2.95		
Exercise + air	331.15 ± 2.53^c^			19	323.06 ± 2.88		
Potassium (mmol l^−1^)							
No exercise	2.42 ± 0.19	1.4	0.25	13	1.74 ± 0.18	0.88	0.42
Exercise	2.08 ± 0.27			16	2.14 ± 0.21		
Exercise + air	1.69 ± 0.20			19	2.43 ± 0.29		
Sodium (mmol l^−1^)							
No exercise	156.70 ± 1.41	1.3	0.27	13	160.17 ± 1.59	0.034	0.97
Exercise	161.49 ± 1.71			16	159.83 ± 1.54		
Exercise + air	163.10 ± 1.60			19	161.16 ± 1.84		
Mean cell haemoglobin concentration (g l^−1^)							
No exercise	255.23 ± 4.56^a^	4.9	**0.0099**	13	245.21 ± 4.89	0.74	0.48
Exercise	238.10 ± 4.41^b^			16	244.98 ± 4.86		
Exercise + air	230.82 ± 4.74^b^			19	233.49 ± 4.58		
Ventilation rate post-treatment (breaths min^−1^)							
No exercise	104.44 ± 2.21^a^	4.7	**0.012**	13	87.39 ± 2.51	0.33	0.72
Exercise	96.85 ± 2.86^a^			16	97.19 ± 3.09		
Exercise + air	79.48 ± 2.63^b^			19	98.07 ± 3.71		
Haematocrit (%)							
No exercise	35.00 ± 1.16	0.47	0.63	13	36.61 ± 1.16	2.0	0.14
Exercise	37.36 ± 1.38			16	36.44 ± 1.51		
Exercise + air	40.23 ± 1.37			19	39.68 ± 1.34		
Haemoglobin (g l^−1^)							
No exercise	88.42 ± 2.52	1.5	0.23	13	88.97 ± 2.43	0.20	0.82
Exercise	88.14 ± 2.81			16	87.94 ± 3.15		
Exercise + air	91.96 ± 2.77			19	91.73 ± 2.44		

The *F* and *P* values for two-way ANOVA are presented (*n* = 32 ± 1 for treatment groups and *n* = 33 ± 4 for temperature groups), with bold text indicating significance after false-discovery rate correction for multiple comparisons. Significant differences between groups were evaluated *post hoc* with Welch's *t*-tests (also see Results section), and are indicated by different superscript letters.

### Survival analysis

**Figure 2:**
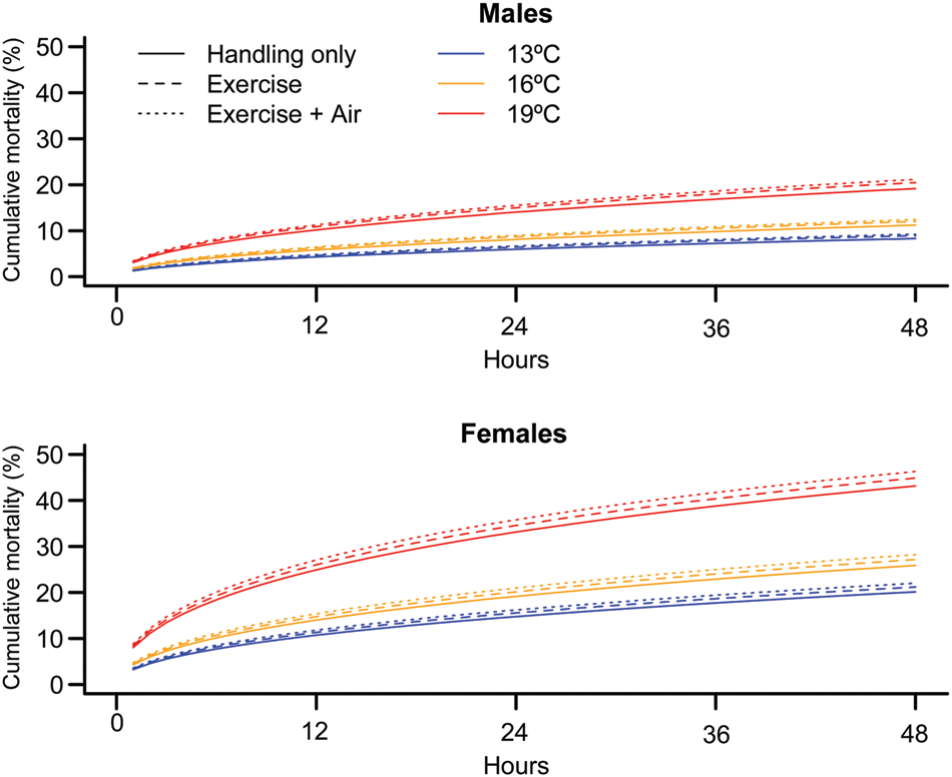
Curves of cumulative mortality over time (48 h) of female and male sockeye salmon after simulated capture–release treatment. The mortality curves are model-averaged estimates based on the 95% confidence set for the best model (see Table 2) that were calculated to account for model selection uncertainty. Blue lines indicate 13°C, yellow lines 16°C and red lines 19°C temperature treatments. Line style indicates the simulated capture–release treatment.

Forty-eight hours after capture–release, the greatest mortality was observed in the 19°C groups exposed to capture–release with and without air exposure (four fish or 40% in each group). No mortality was observed in the 16°C capture–release group in the first 48 h. Intermediate mortality was observed in the 13°C control group (one fish or 7%), the 13°C capture–release air-exposed group (one fish or 9%), the 16°C control and capture–release air-exposed group (three fish or 30% each), the 19°C control group (three fish or 33%) and the 13°C capture–release group (four fish or 36%). The most parsimonious model describing the cumulative mortality data to 48 h included the effects of temperature and sex (Table 2). Similar models with various combinations of capture–release treatment, temperature and sex (and their interactions) were weakly supported by the data (i.e. delta.AICc >3). Model-averaged mortality estimates (and observed mortality) 48 h after treatment were greater for females than males, and were greater at 19°C than at 13 and 16°C for both sexes (Fig. 2). Most of the mortality occurred within the first 24 h.

**Table 2:** Experimental factors of the top eight models (95% confidence set) predicting 48 h mortality of experimental sockeye salmon

Model	*K*	AICc	delta.AICc	weight.AICc	Log likelihood
Temperature + sex	5	397.695	0.000	0.540	−193.518
Sex	3	400.698	3.003	0.120	−197.220
Temperature + treatment + sex	7	400.698	3.004	0.120	−192.720
Temperature × sex	7	401.782	4.087	0.070	−193.262
Treatment + sex	5	403.046	5.352	0.037	−196.193
Temperature	4	404.013	6.318	0.023	−197.789
Temperature + treatment × sex	9	404.117	6.422	0.022	−192.024
(no effects)	2	404.535	6.841	0.018	−200.204

Models are ranked by increasing order of the bias-corrected Akaike information criterion (AICc) value, and the model with the lowest AICc is the most parsimonious one describing the data; delta.AICc is the difference in AICc between a given model and the top-ranked model; weight.AICc gives the probability of a given model in the set being the most parsimonious one describing the data; and *K* is the number of parameters in the model.

### Predicting mortality using physiological indices

**Figure 3:**
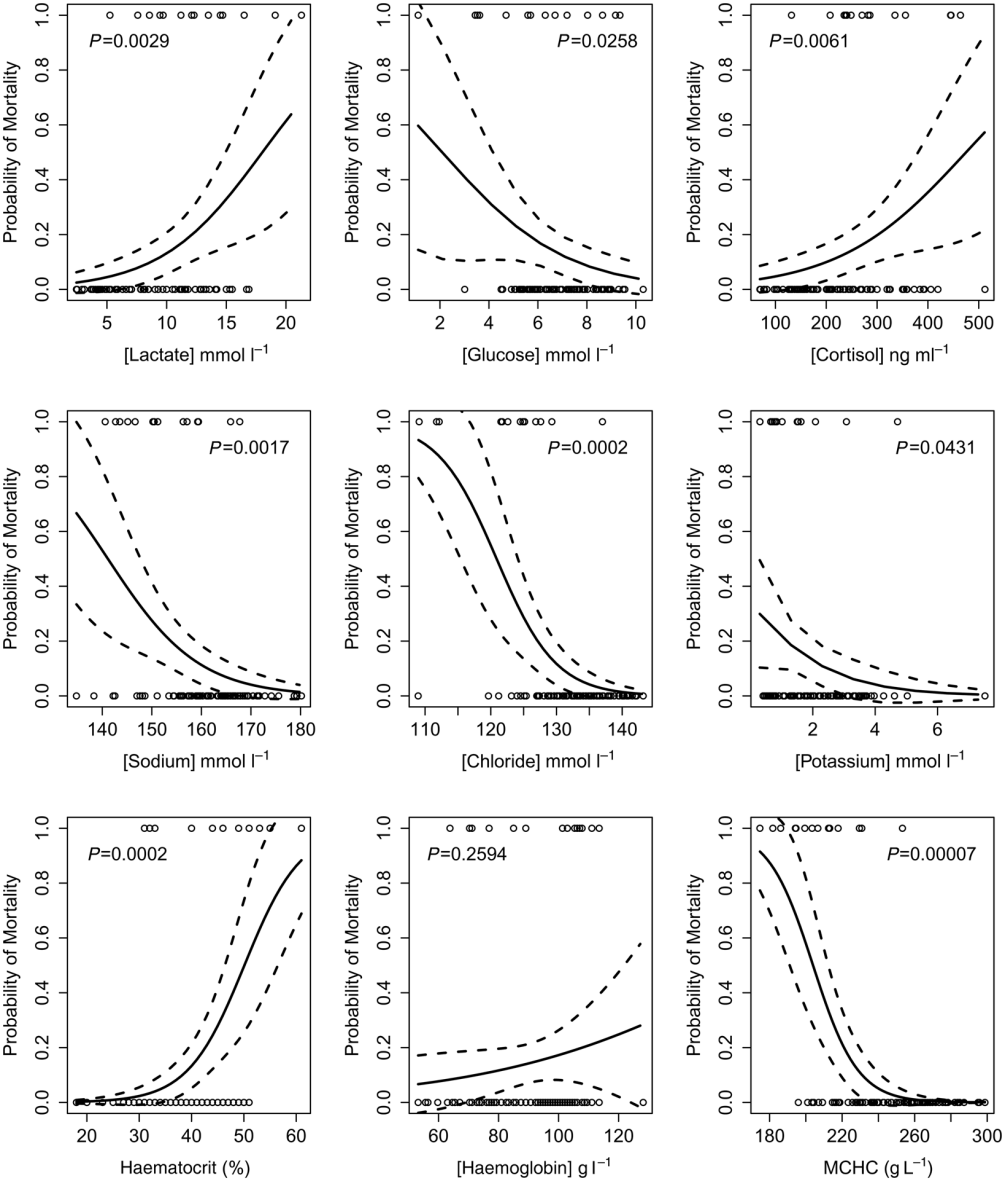
Fitted logistic regression curves of 24 h mortality as a function of blood and plasma parameters measured after capture–release treatment. The logistic regression was conducted for each variable separately and did not take into account capture–release treatment. Continous and dashed lines denote, respectively, mortality estimates and 95% confidence intervals. Open circles denote observed fate of the fish after 24 h, with zero denoting survival and one denoting mortality. All models except that for haemoglobin are significant when evaluated at a critical level corrected for multiple testing.

**Figure 4:**
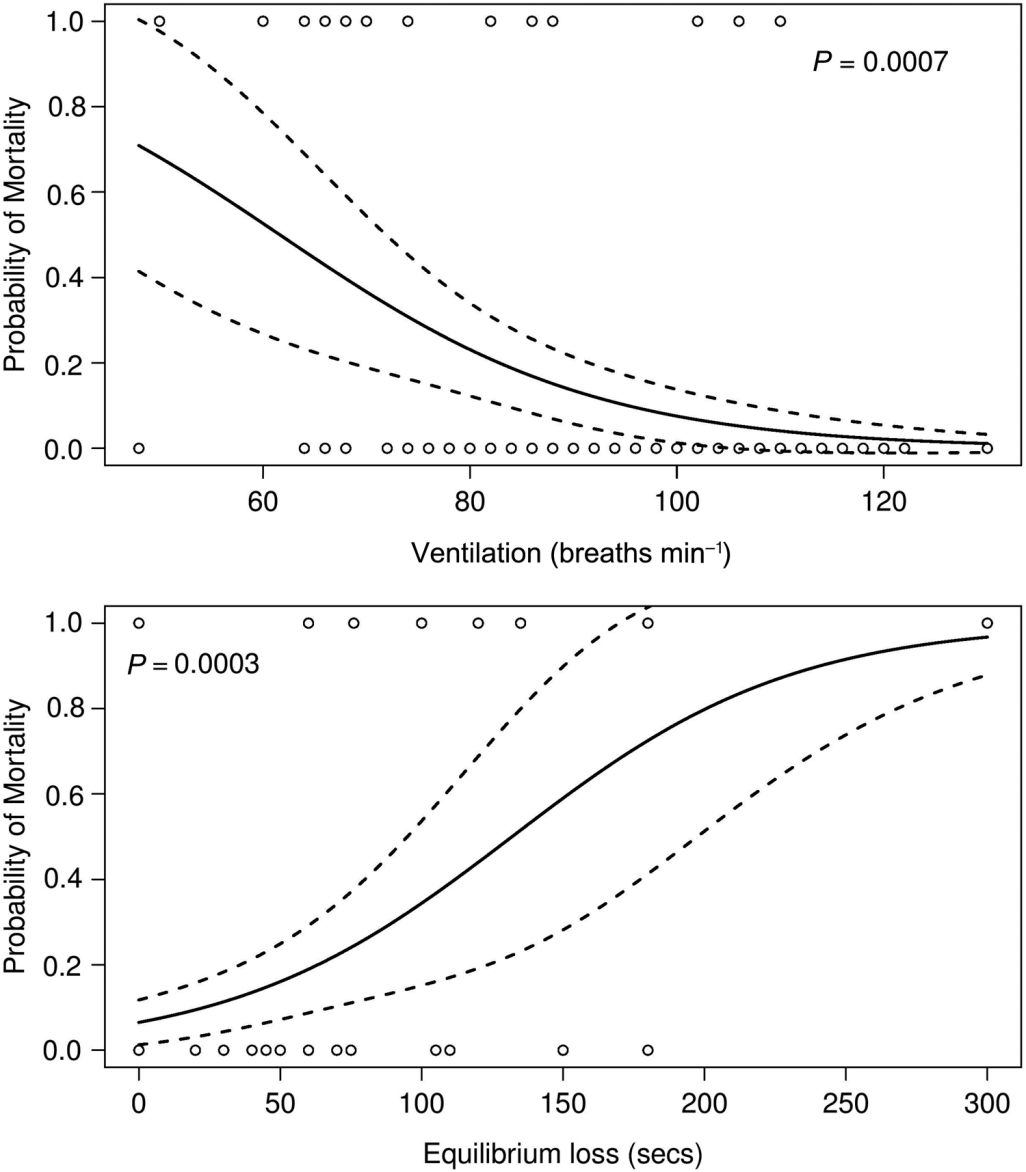
Fitted logistic regression curves of 24 h mortality as a function of ventilation rate (top) and duration of equilibrium loss (bottom) measured after capture–release treatment. The logistic regression was conducted for each variable separately and did not take into account capture–release treatment. Continuous and dashed lines denote, respectively, mortality estimates and 95% confidence intervals. Open circles denote observed fate of the fish after 24 h, with zero denoting survival and one denoting mortality. Both models are significant when evaluated at a critical level corrected for multiple testing.

Logistic regression demonstrated that higher lactate, cortisol and haematocrit, and lower glucose, sodium, chloride, potassium and mean cell haemoglobin concentrations 30 min after capture–release treatment significantly predicted mortality to 24 h (Fig. 3). Likewise, slower ventilation rates and more prolonged equilibrium loss after capture–release treatment significantly increased the probability of mortality to 24 h (logistic regression; Fig. 4). Fish that regained equilibrium in <130s or fish that were ventilating at >62 breaths min^−1^ after release had a >50% probability of surviving 24 h after treatment (Fig. 4).

## Discussion

Annually, tens to hundreds of thousands of Fraser River sockeye salmon are intentionally released from angling gear (DFO, 2010b), and even more encounter net gears and escape by their own struggle. The demonstrable impacts on late-run salmon physiology and survival, from the simulated capture–release scenarios described herein, can be used to help reduce the current uncertainty regarding the fitness consequences to these non-retained sockeye salmon from in-river fisheries at different temperatures. This study is the first to examine how blood chemistry and observable physiological indices, such as equilibrium loss and ventilation rates, can be predictive of mortality after capture and release of sockeye salmon, and it is one of the few studies to investigate the combined effects of capture–release stress, thermal stress and air exposure on Pacific salmon post-release survival (e.g. an associated study examining summer-run sockeye salmon; Gale *et al.*, 2011).

As predicted, ventilation rate and equilibrium were most severely impaired in fish that experienced exhaustive exercise and air exposure at high temperatures. Our results suggest that air exposure increases mortality risk in sockeye salmon by two different mechanisms. First, air exposure frequently causes fish to lose equilibrium (e.g. Gingerich *et al.*, 2007; Thompson *et al.*, 2008), leaving them more vulnerable to secondary capture or predation (e.g. Danylchuk *et al.*, 2007; Raby *et al.*, 2014). This is a considerable risk in the Fraser River, where commercial, First Nations and recreational fishers are prolific, and large populations of predators (i.e. harbour seals, bears and raptors) target returning salmon. Second, air exposure was also associated with depressed ventilation rates (as described by Gale *et al.*, 2011), which can cause decreased respiratory gas exchange; oxygen deprivation by air exposure could result in an impaired ability to correct oxygen deficits incurred during anaerobic exercise. Our results further confirmed the findings of Gale *et al.* (2011) in that elevated temperatures resulted in greater ventilatory impairment in sockeye salmon, which is particularly troubling considering that excess post-exercise oxygen consumption is greatest for fish in warm water (Lee *et al.*, 2003). Elevated temperatures, even in the absence of air exposure, also increased the duration of equilibrium loss (Gale *et al.*, 2011). Despite these physiological impairments, we were not able to demonstrate that greater capture–release stressors directly contributed to lower survival.

We were able to show that the risk of mortality was related to physiological measures of individual fish made after capture–release, and these relationships suggest that it is possible to judge whether captured fish should be released. In order for mortality predictions to be practical for use by fishers, they must involve easily observable metrics, with clear thresholds for established unacceptable risk levels. Ventilatory frequency and duration of equilibrium loss are both easy to observe and quantify, and require no specific expertise to evaluate. Therefore, managers could recommend or mandate (notwithstanding some potential compliance and enforcement challenges) the release of fish above a specific threshold probability of survival and the retention of any fish that had a lesser probability of survival. These types of guidelines could aid fishers in making decisions that would best maintain a healthy fish population. For example, we found that fish that regained equilibrium in <130s or fish that were ventilating at a rate >62 breaths min^−1^ after release had a >50% probability of surviving 24 h after treatment. A simple 2 min rule of thumb for equilibrium loss (or 1 breath s^−1^ rule of thumb for ventilation rate) could be used to infer >50% 24 h survival after release. Interestingly, these impairments were transient for most fish (30 of the 32 fish that lost dorsoventral equilibrium after capture–release appeared recovered within 300 s of release, and ventilation rates had returned to pre-capture levels by the next evaluation 30 min post-capture), but were still predictive of 24 h mortality. Management agencies frequently recommend holding fish until they have recovered orientation and swimming ability, presumably to protect them while they are particularly vulnerable to capture by predators. This research suggests that managers need to be cognizant that fish that are more substantially impaired prior to release (even if recovered to a vigorous state) will still have higher post-release mortality than fish that recover quickly or fish that were not impaired at all.

We successfully used manual chasing with and without air exposure to elicit a physiological stress response similar to that experienced by sockeye salmon migrants encountering fishing gear (Young *et al.*, 2006; Donaldson *et al.*, 2010a), while eliminating the physical injury incurred by these encounters. By omitting injuries sometimes incurred from hooks or net entanglement from our protocols, we were able to evaluate how physiological disturbances alone may affect survival. Our capture–release treatment resulted in 74% higher plasma lactate than in non-capture–release-treated fish, while air-exposed fish had lactate levels 120% higher than non-capture–release-treated fish. Lactate build-ups occurred due to increased glycolytic flux and a corresponding lactacidosis. These results are consistent with other experiments on Pacific salmon using identical capture–release simulation techniques (Donaldson *et al.*, 2010b; Gale *et al.*, 2011). Capture–release treatment resulted in a significant decrease of MCHC compared with non-capture–release-treated fish (driven by a non-significant elevation in haematocrit, with no associated changes in haemoglobin concentration). This is likely to be due to erythrocytic swelling secondary to the adrenaline response resulting from capture stress (Nikinmaa, 1982). Also consistent with our previously published work using simulated capture–release on summer-run sockeye salmon (Gale *et al.*, 2011), we detected no effects of temperature on the blood chemistry parameters measured 30 min after capture–release treatment.

Consistent with our hypothesis, temperatures approaching the critical maximum increased catch-and-release mortality; however, we were surprised to find that sex was a more significant predictor of survival to 48 h than was capture–release treatment. While mortality 48 hours post-treatment was highest in the 19°C air-exposed group, the survival model including temperature and sex was a far more parsimonious fit than any of the models including capture–release treatment. Our finding that females suffered higher mortality than males was consistent with other studies on sockeye salmon, both wild migrants and captured individuals held in laboratory conditions (Crossin *et al.*, 2008; Cooperman *et al.*, 2010; Jeffries *et al.*, 2012). These results suggest that adult females experiencing secondary stressors may be at greater risk of failing to complete their migration and successfully spawn, which could have substantial repercussions on fitness. The lack of significant capture–release treatment effect indicates that capture–release involving a brief strenuous exercise and air exposure may not significantly increase the risk of mortality for sockeye salmon over and above handling alone. However, our finding that the physiological impairments resulting from our simulated capture–release treatments were associated with higher mortality risk suggests that sub-lethal impacts also need to be considered. Studies of capture–release on other species have shown that capture–release stressors and air exposure frequently cause elevations in blood chemistry stress parameters and result in behavioural and other impairments (e.g. Ferguson and Tufts, 1992; Davis and Parker, 2004; Arlinghaus and Hallermann, 2007). In order to understand female mortality of released or escaped sockeye salmon better, researchers could focus on the interaction of temperature and capture–release stress on females specifically.

Generally, capture–release mortality increases at warmer temperatures (reviewed by Gale *et al.*, 2013); however, the present study is the first to show how thermal and capture–release stress may combine to increase mortality risk to released or escaped sockeye salmon. The consequences of this finding with regard to managing sockeye salmon and other fisheries are troubling in a climate-warming scenario. Recent research has shown that sockeye salmon in the Fraser River may be adapted to survive and perform optimally at a narrow range of water temperatures coinciding with historical averages for each genetically and geographically distinct population (Farrell *et al.*, 2008; Eliason *et al.*, 2011). Sockeye salmon are already experiencing temperatures that often exceed their critical thermal maximum in the Fraser River (Patterson *et al.*, 2007; Eliason *et al.*, 2011), which is associated with a high level of migration mortality (Cooke *et al.*, 2004; Hinch and Martins, 2011). Moreover, the Fraser River is expected to continue on this warming trajectory into the future (Ferrari *et al.*, 2007), and this is likely to have consequences for future viability of this valued group of fish (Hague *et al.*, 2011; Martins *et al.*, 2011; Reed *et al.*, 2011). Resource managers are very limited in their ability to stop the increase in water temperature, but they can regulate or make recommendations regarding the other two stressors that we examined, i.e. capture–release stress and air exposure.

Our finding that individual blood and plasma chemistry indicators can predict survival is a promising step towards improving our understanding of post-release mortality for sockeye salmon. Elevated plasma lactate, cortisol, sodium and chloride are consistent with elevated stress (Wendelaar Bonga, 1997), and were all associated with higher probability of mortality within the first 24 h after capture–release. Lactate anions enter the blood from the muscle tissue after anaerobic exercise and are associated with intracellular acidosis. Extreme intracellular acidosis has been suggested to be a causal factor in fish dying after exercise (Wood *et al.*, 1983). Our logistic regression model suggested that sockeye salmon had a >50% probability of mortality within 24 h when plasma lactate concentrations exceeded 18 mmol l^−1^. Migrating sockeye salmon caught by tangle net (Donaldson *et al.*, 2010a), purse seine (Cooke *et al.*, 2008) or beach seine (Clark *et al.*, 2010) may experience similar elevated plasma lactate levels. We detected an association between decreased plasma glucose concentrations and increased mortality risk, which was surprising because blood glucose levels generally increase with stress (Wendelaar Bonga, 1997). We propose that rather than indicating a less-stressed state, the lower glucose concentration could be indicative of fish in poor condition mounting a reduced stress response. An alternative explanation is that severe exercise and/or stress can lead to hepatic glycogen depletion and associated declines in plasma glucose (hypoglycaemia) such as those observed here, particularly over longer time periods (Polakof *et al.*, 2010). The trend of higher haematocrit but equal haemoglobin in capture–release-treated individuals suggests that the depressed MCHC may be a result of erythrocytic swelling. On average, plasma chloride and sodium concentrations 30 min after treatment were similar to conspecifics sampled after river capture (Clark *et al.*, 2010) and ∼15–20% higher compared with quiescent sockeye salmon held in laboratory conditions (Sandblom *et al.*, 2009). Our logistic regression models found that fish with relatively low sodium and chloride ion concentrations 30 min after treatment had a lower probability of surviving 24 h. This is consistent with other research on sockeye salmon demonstrating that chloride values below 120 mmol l^−1^ were associated with increased mortality (Jeffries *et al.*, 2011). Overall, it appears that fish responded physiologically to our treatment protocols in a similar manner to wild migrants caught in various gears, and that several physiological parameters consistent with a generalized stress response were predictive of mortality. In general, the patterns in blood parameters following capture–release were consistent with field results, suggesting the potential transferability of these results to real fisheries, and demonstrating the value of controlled laboratory experiments (Cooke *et al.*, 2013).

Researchers have begun developing novel ways to predict the survival of released fish, perhaps the most promising of which are reflex impairment indices (Davis and Ottmar, 2006; Davis, 2007, 2010). Our study is one of the first to examine how similar observed impairment metrics can be used in Pacific salmon in order to predict mortality (for another example, see Raby *et al.*, 2012). We found that both duration of equilibrium loss and ventilation rate after capture–release treatment were highly significant mortality predictors. Ventilatory frequency has been used in other species to indicate and measure stress responses (e.g. Mock and Peters, 1990; White *et al.*, 2008); however, some caution the utility of this metric because it may not reflect the severity of the stressor (Barreto and Volpato, 2004). Nonetheless, our experiment showed that extreme stressors caused a depression in ventilation rate that was highly predictive of same-day fate in sockeye salmon.

In conclusion, the present study demonstrates that while individual sockeye salmon vary in their responses to simulated capture–release stressors, exhaustive exercise coupled with air exposure at high temperatures can result in a greater mortality risk for released fish than for those not exposed to simulated capture–release. Females were particularly sensitive to stressors, demonstrating the need for management strategies that acknowledge and address inter-sexual variation (Hanson *et al.*, 2008). A better understanding of the physiological predictors of capture–release mortality of sockeye salmon at different temperatures can be used to inform management of the potential consequences of different management actions. Accurate release mortality estimates in various thermal conditions could be applied to total mortality estimates for Fraser sockeye and increase the probability of achieving spawning escapement goals.

## Funding

This work was supported by the University of British Columbia, and by Natural Sciences and Engineering Research Council of Canada (NSERC) Strategic and Discovery Grants to S.G.H. and S.J.C. M.K.G. was supported by an NSERC CGS-M scholarship, the Kathleen and Sheldon Rothwell Fellowship, the Mary and David MacAree Fellowship and the Faculty of Forestry (UBC). S.J.C. is also supported by the Canada Research Chairs program.
